# Neonatal and developmental outcomes of very preterm twins according to the chorionicity and weight discordance

**DOI:** 10.1038/s41598-023-33428-0

**Published:** 2023-04-26

**Authors:** Soo Yeon Lim, Seung Han Shin, Hyo Ju Yang, Seul Gi Park, Ee-Kyung Kim, Han-Suk Kim, Jong Kwan Jun

**Affiliations:** 1 Department of Pediatrics, Seoul National University Bundang Hospital, Seongnam, Republic of Korea; 2 Department of Pediatrics, Seoul National University Children’s Hospital, Seoul, Republic of Korea; 3 Department of Pediatrics, Seoul National University College of Medicine, 101, Daehak-Ro, Jongno-Gu, Seoul, Republic of Korea; 4 Department of Obstetrics & Gynecology, Seoul National University College of Medicine, Seoul, Republic of Korea

**Keywords:** Medical research, Reproductive signs and symptoms

## Abstract

Perinatal outcomes of twin pregnancies are determined by several factors, such as gestational age (GA), chorionicity, and discordance at birth. This retrospective study aimed to investigate the association of chorionicity and discordance with neonatal and neurodevelopmental outcomes in preterm twin infants from uncomplicated pregnancy. Data of very preterm twin infants who were both live-born between 2014 and 2019 on the chorionicity of the twin, diagnosis of the twin-to-twin syndrome (TTTS), weight discordance at birth, and neonatal and neurodevelopmental outcomes at 24 months of corrected age (CA) were collected. Of the 204 twin infants analyzed, 136 were dichorionic (DC) and 68 were monochorionic (MC), including 15 pairs with TTTS. After adjusting for GA, brain injury, including severe intraventricular hemorrhage and periventricular leukomalacia, was mostly found in the MC with TTTS group, with a higher incidence of cerebral palsy and motor delay at CA 24 months. After excluding TTTS, multivariable analysis showed no association between chorionicity and neonatal and developmental outcomes, whereas small infants among co-twins (adjusted odds ratio (aOR) 3.33, 95% confidence interval 1.03–10.74) and greater discordance (%) of weight at birth (aOR 1.04, 1.00–1.07) were associated with neurodevelopmental impairment. Monochorionicity might not determine adverse outcomes among very preterm twins from uncomplicated pregnancy.

## Introduction

The increased use of assisted reproductive technologies and childbearing at an older age has contributed to the increasing trend of multiple births^1^. Therefore, perinatal mortality and morbidity are 3–7 times higher in twin pregnancies than in singleton pregnancies^1,2^. Moreover, a twin pregnancy is associated with adverse postnatal outcomes in preterm infants^3,4^.

There are several alleged factors predicting adverse outcomes in twin pregnancy. Monochorionic (MC) twins have been reported as predictors of perinatal morbidity and mortality^2,5,6^. Vascular anastomoses among MC twins might cause unequal distribution of placental blood flow, which eventually could lead to a certain range of pregnancy complications, including twin-to-twin syndrome (TTTS) and intrauterine death (IUD) of co-twins^7^. However, the effect of monochorionicity on neonatal and developmental outcomes in preterm twins remains controversial. Two single-center studies from South Korea and Brazil reported no differences in neonatal morbidities between MC and dichorionic (DC) twins^6,8^. Conversely, two population-based studies reported a higher incidence of neonatal morbidities and mortalities in MC twins than in DC twins^2,5^. Adverse outcomes in morbidities and neurodevelopment might be attributed to TTTS, discordant weight at birth among pairs, and in utero death of the co-twin^9^. However, as most twin pregnancies do not experience these complications, evaluating the prognosis of MC twins without complicated conditions, such as IUD and TTTS, might be valuable compared with DC twins^10,11^.

Another determinant of postnatal outcomes among twin infants is weight discordance among co-twin^12–14^. The prevalence of discordant twins varies according to the definition of weight discordance at birth and has been reported to be approximately 30%^8,12^. Discordance among twin infants can occur in both MC and DC twins; however, the pathogenesis of weight discordance differs according to chorionicity. Different genetic backgrounds and placental insufficiency have been suggested to lead to weight discordance in DC twins. In contrast, placental vascular anastomoses with hemodynamic imbalance are commonly postulated as the cause of growth discordance in MC twins^11,15^. As discordant twins are more prevalent in MC twins^6^ and are also associated with adverse outcomes in twin infants, adjusting for these two factors or confining the study population might help elucidate the role of these two factors on the neonatal and developmental outcomes of twin infants.

Studies on twin pregnancies have mainly focused on pregnancy outcomes, including perinatal mortality and IUD, and the study population has been term and late preterm infants^2,6,8,9,12^. However, data on the neurodevelopmental outcomes of very preterm infants born from twin pregnancies are lacking. Therefore, this study aimed to evaluate the neonatal and neurodevelopmental outcomes of very preterm twin infants who were both live-born and to investigate which is a significant determinant for the adverse neurodevelopmental outcome between chorionicity and weight discordance.

## Methods

### Study design and participants of the study

This retrospective cohort study included preterm infants who were delivered from twin gestations and were both live-born at < 32 weeks at Seoul National University Hospital between January 2014 and December 2019. We excluded infants with chromosomal abnormalities from the study. The study population was categorized as MC and DC twins based on chorionicity, determined by ultrasonography according to the number of gestational sacs or the presence of lambda or T-sign^16^. Among the MC twins, twin-to-twin transfusion syndrome (TTTS) was further categorized and diagnosed prenatally based on the presence of a monochorionic diamniotic pregnancy with oligohydramnios and polyhydramnios using the Quintero staging system^17,18^.

### Data collection

We collected data on perinatal characteristics and neonatal outcomes, such as respiratory distress syndrome (RDS), patent ductus arteriosus requiring treatment, necrotizing enterocolitis, and moderate-to-severe bronchopulmonary dysplasia (BPD), from electronic medical records^19,20^. We identified intraventricular hemorrhage grade ≥ 3 and periventricular leukomalacia, diagnosed by brain sonographic study during the neonatal intensive care unit (NICU) stay and/or brain magnetic resonance imaging at term equivalent age, as significant brain injuries^21^. Weight discordance at birth was calculated as a percentage of [(large infant—small infant)/large infant of birth weight], and a difference in birth weight of ≥ 20% was defined as a discordant twin^22^. The neonatal composite outcome was defined as moderate-to-severe BPD, brain injury, or death.

### Assessment of growth and neurodevelopment

Weight and height at birth, discharge, and corrected ages (CA) of 4, 12, 18, and 24 months were reviewed, and the z-scores of each measurement for sex and age were calculated based on the World Health Organization child growth standards and Fenton growth charts^23^. Data on developmental outcomes were collected at the CA of 24 months. Cerebral palsy was defined as a non-progressive central nervous system disorder characterized by abnormal muscle tone in more than one extremity and abnormal control of movement and posture^24^. The results of the Bayley Scales of Infant and Toddler Development 3rd Edition (Bayley-III) were reviewed, and scores < 85 (< − 1 SD) in both the cognitive and language domains or a motor score < 85 were defined as developmental delays^25^. The need for unilateral or bilateral hearing aids was defined as hearing impairment. Any of the following criteria were defined as having a neurodevelopmental impairment (NDI), including cerebral palsy (CP), any delay in Bayley-III, blindness, or hearing impairment^26^. The composite outcomes of NDI and death were compared among the study groups.

### Analytical methods

To compare perinatal characteristics and neonatal and developmental outcomes, we categorized the study’s population as DC twins, MC twins with TTTS, and MC twins without TTTS. Continuous variables were compared using analysis of variance with Bonferroni correction, and categorical variables were compared using Fisher’s exact test, with *p* values for adjusted proportions regarding gestational age (GA). To evaluate the effect of monochorionicity on the outcomes, we excluded pathological conditions of TTTS. Then, we conducted a univariate analysis of GA, sex, MC twin, small infants of each pair, and weight discordance for moderate-to-severe BPD, brain injury, neonatal death, neonatal composite outcome, NDI, and the composite outcome of NDI or death at CA of 24 months. Multiple logistic regression analysis among the DC and MC twins without TTTS included GA, sex, and twin-related factors, such as MC twins, small infants, and weight discordance. As for growth, we compared the z-scores of weight and height between small and large babies of DC and MC twins, excluding TTTS stage IV, until CA of 24 months, and in the subgroup of discordant twins. A statistically significant difference was set at *p* < 0.05.

### Ethics declarations

This study protocol was reviewed and approved by the Institutional Review Board of Seoul National University Hospital (approval No. 2112–118-1284). The need for informed consent was waived by the institutional review board because of the nature of the retrospective study. All methods used in this study were performed in accordance with Declaration of Helsinki.

## Results

### Characteristics of the study population

**Figure 1:**
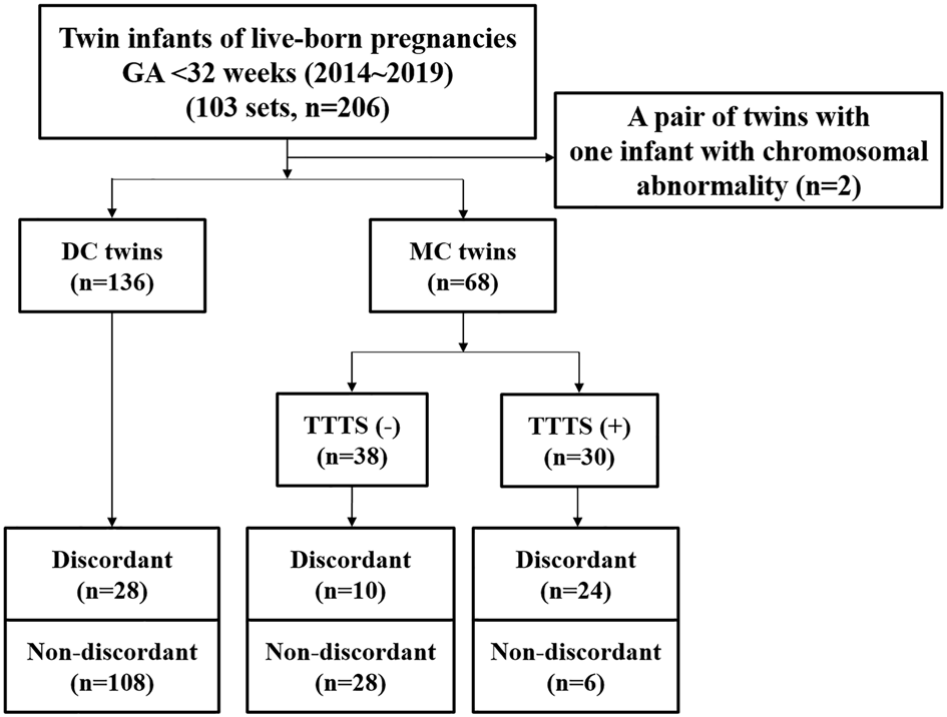
Flow chart of the study population.

Among 206 preterm twin infants (103 pairs) born < 32 weeks during the study period, one pair of twins (n = 2) was excluded because of a chromosomal abnormality (Fig. 1). There were 136 infants with DC twins and 68 with MC twins, including 15 pairs of TTTS. GA of the study population was 29.8 (27.4–31) weeks, and birth weight was 1,215 (935–1,465) grams (Table 1). Overall, 62 (30.4%) and 15 (7.4%) infants had weight discordance and were small for GA, respectively. The prevalence of moderate-to-severe BPD, brain injury, death, and neonatal composite outcome was 21.5%, 14.7%, 13.7%, and 36.8% in the study population, respectively. Among TTTS twins, there were six pairs of stage I, one of stage II, three of stage III, and five of stage IV twins. In addition, three pairs of twins in stage IV and two in stage III were treated with fetal laser photocoagulation. Among the 12 infants with severe TTTS (stages III–IV) and laser photocoagulation, 8 (66.7%) patients had adverse neonatal composite outcomes, and 6 (50%) had a composite outcome of NDI or death at CA 24 months, whereas 2 of the 4 (50%) infants with severe TTTS did not receive laser photocoagulation and had an adverse neonatal composite outcome and 1 (25%) had a composite outcome of NDI or death at CA 24 months. Ad-hoc analysis of MC twins without TTTS, MC twins with mild TTTS (stages I–II), and MC twins with severe TTTS (III–IV) showed that there were no statistical differences in the neonatal and developmental outcomes between mild TTTS and severe TTTS (Table S1).

**Table 1:** Demographic findings of total study population.

	N = 204
Gestational age (week)	29.8 (27.4–31)
Birth weight (g)	1215 (935–1465)
Apgar score 1 min	4 (2–6)
Apgar score 5 min	7 (6–8)
Pregnancy-induced hypertension	6 (3)
Preterm premature rupture of membrane	91 (45.1)
Histologic chorioamnionitis	86 (42.8)
Oligohydramnios	50 (24.8)
Cesarean section	108 (52.9)
Prenatal steroid	186 (91.2)
Male	102 (50)
Small for gestational age	15 (7.4)
Monochorionic twin	68 (33.3)
Discordant twin	62 (30.4)
Twin-to-twin transfusion syndrome	30 (14.7)
Neonatal outcomes	
Respiratory distress syndrome	112 (55.5)
Moderate-to-severe BPD	38 (21.5)
Necrotizing enterocolitis	10 (4.9)
*Brain injury	30 (14.7)
Sepsis	32 (16.3)
Death	28 (13.7)
BPD or brain injury or death	75 (36.8)
At the corrected age of 24 months	
Cerebral palsy	8 (4.7)
Blindness	0 (0)
Hearing impairment	3 (1.8)
Bayley-III (n = 131)	
Cognitive	100 (90–105)
Language	97 (89–106)
Motor	100 (91–103)
Any delay	19 (14.5)
NDI	25 (14.6)
Composite outcome of NDI or death	53 (26.6)

Values are expressed as N (%) or median (interquartile range). *BPD* bronchopulmonary dysplasia; Bayley-III, Bayley scales of infant and toddler development 3rd edition, *NDI* Neurodevelopmental impairment. *Brain injury was defined as an intraventricular hemorrhage of grade ≥ 3 or periventricular leukomalacia.

### Neonatal and developmental outcomes

GA was lower in DC twins than in MC twins without TTTS (29.2 vs. 30.3 weeks), and Apgar scores at 1 and 5 min were lowest in MC twins with TTTS (Table 2). Preterm premature rupture of membrane and histological chorioamnionitis were mostly found in the DC twin group; conversely, oligohydramnios and discordant twins were most prevalent in MC twins with TTTS. RDS and brain injury incidence were the highest in MC twins with TTTS (78.6% and 30%, respectively), with the highest incidence of PVL (28.6%) (Table 2). In contrast, the incidence of moderate-to-severe BPD was lowest in MC twins without TTTS (6.3%). Adverse neonatal composite outcomes of BPD or brain injury or death were more prevalent in the MC-TTTS ( +) group than in the MC-TTTS ( −) group. After adjusting for GA, RDS, PVL, and brain injury were mostly found in the MC with TTTS group. The difference in the incidence of moderate-to-severe BPD and the neonatal composite outcome became insignificant.

**Table 2:** Demographic findings, neonatal and developmental outcomes according to the study group.

	DC twin(n = 136)	MC twin		*p* value	Adjusted *p* value
		MC-TTTS ( −) (n = 38)	MC-TTTS ( +)\ (n = 30)		
Gestational age (week)	29.2 (26.4–31)	30.3 (29–31)	29 (26.9–30.6)	0.011^§§^	
Birth weight (g)	1205 (880–1460)	1300 (1080–1490)	1170 (910–1390)	0.108	
Apgar score 1 min	4 (2–6)	5 (4–7)	3 (2–4)	< 0.001*^,^**	
Apgar score 5 min	7 (6–8)	7 (7–8)	6 (4–7)	< 0.001*^,^**	
PIH	6 (4.4)	0 (0)	0 (0)	0.361	
PPROM	78 (57.4)	11 (29)	2 (7.1)	< 0.001^§§,^*	
hCAM	71 (52.6)	7 (18.4)	8 (28.6)	< 0.001^§§^	
Oligohydramnios	33 (24.3)	3 (7.9)	14 (50)	0.001*^,^**	
Cesarean section	52 (38.2)	30 (79)	26 (86.7)	< 0.001^§§,^*	
Prenatal steroid	128 (94.1)	36 (94.7)	22 (73.3)	0.003*	
Male	75 (55.2)	15 (39.5)	12 (40)	0.123	
SGA	9 (6.6)	2 (5.3)	4 (13.3)	0.396	
Discordant twin	28 (20.6)	10 (26.3)	24 (80)	< 0.001*^,^**	
Neonatal outcomes					
RDS	76 (55.9)	14 (36.8)	22 (78.6)	0.003**	0.010
Mod to severe BPD	31 (26.3)	2 (6.3)	5 (18.5)	0.037^§§^	0.275
NEC	8 (5.9)	1 (2.6)	1 (3.3)	0.888	0.956
IVH ≥ grade III	14 (10.8)	1 (2.7)	2 (7.1)	0.321	0.946
PVL	9 (7.3)	1 (3)	8 (28.6)	0.003*^,^**	0.002
Brain injury	19 (14)	2 (5.3)	9 (30)	0.019**	0.026
Sepsis	26 (19.7)	3 (8.3)	3 (10.7)	0.217	0.860
Death	21 (15.4)	4 (10.5)	3 (10)	0.714	0.345
BPD or brain injury or death	53 (39)	7 (18.4)	15 (50)	0.015**	0.240
At the corrected age of 24 months					
Cerebral palsy	3 (2.7)	1 (3.1)	4 (14.8)	0.040	0.032
Hearing impairment	2 (1.8)	0 (0)	1 (3.7)	0.477	0.510
Bayley-III	n = 91	n = 20	n = 20		
Cognitive	100 (90–110)	95 (90–105)	100 (92.5–107.5)	0.903	0.532
Language	94 (89–106)	97 (87.5–106)	98.5 (90–113.5)	0.724	0.577
Motor	100 (91–103)	100 (94–107)	89.5 (79–103)	0.004*^,^**	0.002
Any delay	12 (13.2)	2 (10)	5 (25)	0.326	0.340
NDI	15 (13.4)	3 (9.4)	7 (25.9)	0.181	0.263
Composite outcome of NDI or death	36 (27.1)	7 (19.4)	10 (33.3)	0.445	0.406

Values are expressed as N (%) or median (interquartile range). ^§^Adjusted for gestational age at birth; ^§§^ for significant difference between DC twin and MC-TTTS ( −), * for significant difference between DC twin and MC-TTTS ( +), and ** for significant difference between MC-TTTS ( −) and MC-TTTS ( +) using Bonferroni correction or ad-hoc analysis (p < 0.017). *DC* Dichorionic, *MC* Monochorionic, *TTTS* Twin-to-twin transfusion syndrome, *PIH* Pregnancy-induced hypertension, *PROM* Preterm premature rupture of membrane, *hCAM* Histologic chorioamnionitis, *SGA* Small for gestational age, *RDS* Respiratory distress syndrome, *BPD* Bronchopulmonary dysplasia, *NEC* Necrotizing enterocolitis, Bayley-III, Bayley scales of infant and toddler development 3rd edition, *NDI* Neurodevelopmental impairment. Brain injury was defined as an intraventricular hemorrhage of grade ≥ 3 or periventricular leukomalacia.

At CA of 24 months, CP occurred more frequently in the MC with TTTS group, with the lowest score in the motor domain of Bayley-III among study groups. However, no cases of blindness were found, and the incidence of hearing impairment was comparable among the study groups. Consequently, there were no differences in the NDI or composite outcome of NDI or death at CA 24 months among the DC twin group, MC twin without TTTS group, and MC twin with TTTS group.

### Effects of chorionicity and weight discordance on the outcomes

Multiple logistic regression analysis of the study population excluding MC twins with TTTS showed that GA was associated with neonatal morbidity and mortality, as well as NDI and the composite outcome of NDI or death at 24 months’ CA (Table 3). MC twins were not associated with morbidities, mortality, NDI, or composite outcomes. Small infants were at higher risk of moderate-to-severe BPD (adjusted odds ratio (aOR) 3.94, 95% confidence interval (CI) 1.33–11.66), neonatal composite outcome (aOR 3.66, 95% CI 1.42–9.46), and NDI (aOR 3.33, 95% CI 1.03–10.74). Greater discordance in weight at birth was also associated with moderate-to-severe BPD (aOR 1.04, 95% CI 1.00–1.07), NDI (aOR 1.04, 95% CI 1.00 –1.07), or composite outcome of NDI or death (aOR 1.03, 95% CI 1.00–1.05).

**Table 3:** Association of twin-related factors with neonatal and neurodevelopmental outcomes for the study population without TTTS.

Outcomes	Twin associated factor	^§^Adjusted OR (95% CI)	*p* value
Moderate-to-severe BPD	GA (week)	0.47 (0.35–0.63)	< 0.001
	MC twin	0.52 (0.1–2.63)	0.425
	Small infant	3.94 (1.33–11.66)	0.013
	Weight discordance (%)	1.04 (1.00–1.07)	0.039
*Brain injury	GA (week)	0.59 (0.47–0.74)	< 0.001
	MC twin	1.17 (0.21–6.51)	0.862
	Small infant	0.53 (0.18–1.61)	0.265
	Weight discordance (%)	1.02 (0.98–1.06)	0.329
Neonatal death	GA (week)	0.54 (0.43–0.69)	< 0.001
	MC twin	3.55 (0.8–15.84)	0.097
	Small infant	1.86 (0.65–5.34)	0.250
	Weight discordance (%)	1.02 (0.98–1.06)	0.377
Neonatal composite outcome of BPD or brain injury or death	GA (week)	0.41 (0.32–0.54)	< 0.001
	MC twin	1.26 (0.41–3.91)	0.684
	Small infant	3.66 (1.42–9.46)	0.007
	Weight discordance (%)	1.03 (1–1.06)	0.066
NDI at CA of 24 months	GA (week)	0.63 (0.49–0.81)	< 0.001
	MC twin	1.48 (0.34–6.4)	0.600
	Small infant	3.33 (1.03–10.74)	0.044
	Weight discordance (%)	1.04 (1.00–1.07)	0.031
Composite outcome of NDI or death at CA of 24 months	GA (week)	0.58 (0.49–0.69)	< 0.001
	MC twin	1.41 (0.59–3.36)	0.444
	Small infant	1.58 (0.74–3.39)	0.239
	Weight discordance (%)	1.03 (1.00–1.05)	0.033

^§^Adjusted for gestational age, sex, MC twin, small infant, and weight discordance. Multiple logistic regression analysis was conducted among dichorionic and MC twins without twin-to-twin syndrome. *OR* Odds ratio, *BPD* Bronchopulmonary dysplasia, *GA* Gestational age, *MC* Monochorionic, *NDI* neurodevelopmental impairment, *CA* Corrected age. *Brain injury was defined as an intraventricular hemorrhage of grade ≥ 3 or periventricular leukomalacia.

### Growth patterns of discordant DC and MC twins

**Figure 2:**
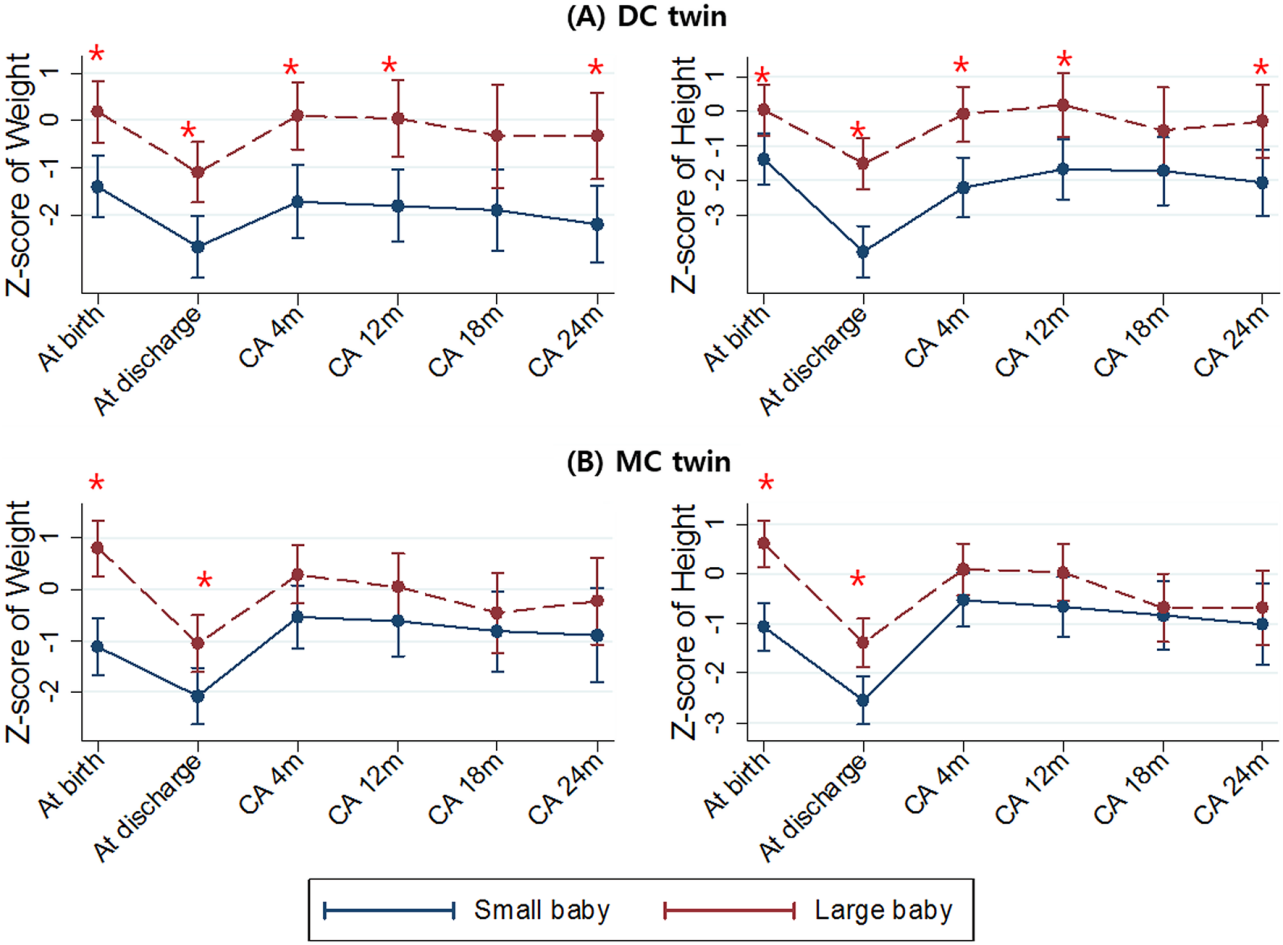
Growth of small and large infants of discordant dichorionic twin (**A**) and discordant monochorionic twin (**B**).CA, corrected age.

During the NICU stay, z-scores of weight and height were significantly higher in the large infant of both DC and MC twins (shown in Fig. S1). Although differences in height z-scores persisted until CA of 4 months in DC twins, differences in weight z-score after discharge disappeared among twin pairs, and differences in height z-score after 4 months of CA disappeared in both DC and MC twins. In the subgroup of discordant twins, differences in z-scores of weight and height persisted until CA of 24 months in DC twins, except at CA 18 months; however, those differences disappeared after discharge in the MC twin (shown in Fig. 2).

## Discussion

In this study of very preterm infants, MC twins with TTTS had more adverse outcomes of RDS, brain injury, and delay in motor development at CA of 24 months than MC twins without TTTS and DC twins. After excluding TTTS, monochorionicity was not associated with neonatal outcomes of moderate-to-severe BPD, brain injury, neonatal death, neonatal composite outcome, or neurodevelopmental impairment at CA of 24 months. Instead, the smaller of the twins and greater weight discordance at birth were associated with moderate-to-severe BPD and neurodevelopmental impairment.

No increase in adverse neonatal outcomes in MC twins has been reported in previous studies^6,8^. Regarding the neurodevelopmental outcomes, two studies reported that intellectual abilities in school-age children or adults were not influenced by monochorionicity or monozygosity^27,28^. Although a few studies have demonstrated a higher incidence of adverse neurodevelopment in MC twins, differences in outcomes were primarily attributed to weight discordance at birth, IUD of co-twins, and TTTS^9,29^. After excluding twins with TTTS during multiple logistic regression analysis, weight discordance at birth was significantly associated with NDI. Therefore, multivariable analysis was necessary to evaluate the role of these two factors in neurodevelopmental outcomes because weight discordance has been proposed to be a significant predictor of neuromorbidity in twin infants^12,30^ and reported to be more prevalent in MC twins than in DC twins^6,31^.

Fetal death in utero has been shown to be higher in MC twins than in DC twins^2,5,6,9^. However, there have been controversies regarding MC and DC twins’ neonatal deaths. In a population-based Dutch study, there was no difference in neonatal death between MC and DC twins^2^. In contrast, neonatal death was more prevalent in DC twins in a single-center retrospective study by Park et al.^6^ and MC twins in a study of registered data from the United Kingdom^5^. This controversy might be attributed to the study population of each study. The Dutch and UK studies included TTTS and twins with IUD of co-twins; however, Park’s study excluded TTTS and twins with IUD in comparing neonatal deaths. Furthermore, Park et al. reported that higher mortality in DC twins was incident, as those who died among the DC twins were extremely preterm infants. In contrast, there were no preterm infants of GA < 30 weeks in MC twins in the study population. This study showed no difference in neonatal mortality between MC and DC twins when we excluded those with IUD of co-twin and TTTS. This is consistent with another twin study excluding TTTS, which reported no difference in neonatal mortality according to chorionicity and weight discordance^8^. Moreover, this study found no association between chorionicity and moderate-to-severe BPD, brain injury, or NDI.

Intriguingly, there were differences in the growth pattern until 2 years of CA between MC and DC twins with weight discordance at birth. Subsequently, differences in the z-scores of weight and height among co-twins became insignificant after discharge in discordant DC twins; however, these differences were still found at 24 months of CA in discordant MC twins. This difference in growth patterns might be attributed to the genetic background of chorionicity in twin infants. Although 30% of monozygotic twins are dichorionic and 10% of dichorionic twins are monozygotic, all monochorionic twins are monozygoticy^32^. Therefore, the similarity in genetic potential in MC twins may attenuate the discordance in growth during the first 2 years of life.

Our study has some limitations. First, the small sample size and retrospective nature of the study design. Second, Bayley-III at CA of 24 months was performed in only 64% of the study population, although other developmental outcomes of CP, hearing problems, and blindness were well documented. Furthermore, the study population was confined to those without IUD of co-twins, and analysis of the association with NDI was restricted to twins without TTTS. Therefore, the results of this study should be interpreted with caution, as we aimed to identify predictors among very preterm twin infants without obstetric complications. Therefore, this result- no association of chorionicity on the neonatal and developmental stages -might be confined to only preterm infants without obstetric complications, such as TTTS and IUD of co-twins. However, this study’s results might be valuable because most twin pregnancies do not experience IUD in co-twins and TTTS. Another strength of this study was that only very preterm infants were included regarding neurodevelopmental outcomes, as the study populations of the studies mentioned above on chorionicity and weight discordance were mostly for late preterm or term and a limited number of very preterm infants. Therefore, this study population’s growth and developmental outcomes may provide informative data for clinicians and researchers.

## Conclusion

In very preterm twin infants who were both live-born and without a history of TTTS, monochorionicity was not associated with adverse neonatal or developmental outcomes. However, smaller infants among co-twins and greater discordance in weight at birth were associated with moderate-to-severe BPD and adverse neurodevelopmental outcomes.

## Supplementary Information

Supplementary Information.

## Data Availability

The datasets used and/or analyzed during the current study are available from the corresponding author on reasonable request.
