# Supplementary material for: Neonatal and developmental outcomes of very preterm twins according to the chorionicity and weight discordance
Source: Sci Rep. 2023 Apr 26;13:6784. doi: 10.1038/s41598-023-33428-0 (PMC10133444; doi:10.1038/s41598-023-33428-0)

**Neonatal and developmental outcomes of very preterm twins according to the chorionicity and weight discordance**

Soo Yeon Lim^1^, *Seung Han Shin^2,3^, Hyo Ju Yang^2^, Seul Gi Park^2,3^, Ee-Kyung Kim^2,3^, Han-Suk Kim^2,3^, Jong Kwan Jun^4^

^1^ Department of Pediatrics, Seoul National University Bundang Hospital, Seongnam, Republic of Korea

^2^ Department of Pediatrics, Seoul National University Children’s Hospital, Seoul, Republic of Korea

^3^ Department of Pediatrics, Seoul National University College of Medicine, Seoul, Republic of Korea

^4^ Department of Obstetrics & Gynecology, Seoul National University College of Medicine, Seoul, Republic of Korea

*Corresponding Author:

Seung Han Shin M.D., Ph.D.

Department of Pediatrics, Seoul National University College of Medicine

101, Daehak-ro, Jongno-gu, Seoul, Republic of Korea

Tel: +82-2-2072-7230

E-mail: revival421@snu.ac.kr

Keywords: chorionicity, weight discordance, preterm twins, monochorionic, dichorionic

Table S1. Demographic findings, neonatal and developmental outcomes among monochorionic twins according to the diagnosis and severity of twin to twin transfusion syndrome

|  | MC-TTTS (-) (n=38) | Mild TTTS (n=14) | Severe TTTS (n=16) |  |
| --- | --- | --- | --- | --- |
| Gestational age (week) | 30.3 (29-31) | 30.4 (27.1-31.3) | 28.6 (26.4-30) | 0.002^*^ |
| Birth weight (g) | 1300 (1080-1490) | 1170 (910-1460) | 1160 (905-1390) | 0.763 |
| Apgar score 1 min | 5 (4-7) | 2 (1-4) | 3.5 (3-4.5) | <0.001^§,*^ |
| Apgar score 5 min | 7 (7-8) | 6 (3-7) | 6 (5-7) | <0.001^§,*^ |
| PPROM | 11 (29) | 0 (0) | 2 (14.3) | 0.048 |
| hCAM | 7 (18.4) | 4 (28.6) | 4 (28.6) | 0.559 |
| Oligohydramnios | 3 (7.9) | 7 (50) | 7 (50) | <0.001^§,*^ |
| Cesarean section | 30 (79) | 14 (100) | 12 (75) | 0.130 |
| Prenatal steroid | 36 (94.7) | 10 (71.4) | 12 (75) | 0.029 |
| Male | 15 (39.5) | 6 (42.9) | 6 (37.5) | 1.000 |
| SGA | 2 (5.3) | 2 (14.3) | 2 (12.5) | 0.453 |
| Discordant twin | 10 (26.3) | 12 (85.7) | 12 (75) | <0.001^§,*^ |
| Neonatal outcomes |  |  |  |  |
| RDS | 14 (36.8) | 10 (71.4) | 12 (85.7) | 0.002^*^ |
| Mod to severe BPD | 2 (6.3) | 2 (14.3) | 3 (23.1) | 0.243 |
| NEC | 1 (2.6) | 1 (7.1) | 0 (0) | 0.424 |
| IVH ≥grade III | 1 (2.7) | 0 (0) | 2 (14.3) | 0.229 |
| PVL | 1 (3) | 4 (28.6) | 4 (28.6) | 0.011 |
| Brain injury | 2 (5.3) | 4 (28.6) | 5 (31.3) | 0.013 |
| Sepsis | 3 (8.3) | 1 (7.1) | 2 (14.3) | 0.846 |
| Death | 4 (10.5) | 0 (0) | 3 (18.8) | 0.277 |
| BPD or brain injury or death | 7 (18.4) | 5 (35.7) | 10 (62.5) | 0.008^*^ |
| At CA 24 months |  |  |  |  |
| Cerebral palsy | 1 (3.1) | 1 (7.1) | 3 (23.1) | 0.053 |
| Hearing impairment | 0 (0) | 1 (7.1) | 0 (0) | 0.482 |
| Bayley-III | n=20 | n=11 | n=9 |  |
| Cognitive | 95 (90-105) | 105 (95-115) | 95 (70-105) | 0.070 |
| Language | 97 (87.5-106) | 97 (89-112) | 100 (97-115) | 0.978 |
| Motor | 100 (94-107) | 91 (85-103) | 85 (58-103) | 0.007^*^ |
| Any delay | 2 (10) | 1 (9.1) | 4 (44.4) | 0.063 |
| NDI | 3 (9.4) | 3 (21.4) | 4 (30.8) | 0.207 |
| Composite outcome of death or NDI | 7 (19.4) | 3 (21.4) | 7 (43.8) | 0.211 |

Values are expressed as N (%) or median (interquartile range). ^§^Adjusted for gestational age at birth; ^§§^ for significant difference between DC twin and MC-TTTS (-), ^*^ for significant difference between DC twin and MC-TTTS (+), and ^**^ for significant difference between MC-TTTS (-) and MC-TTTS (+) using Bonferroni correction or ad-hoc analysis. DC, dichorionic; MC, monochorionic; TTTS, twin-to-twin transfusion syndrome; PROM, preterm premature rupture of membrane; hCAM, histologic chorioamnionitis; SGA, small for gestational age; RDS, respiratory distress syndrome; BPD, bronchopulmonary dysplasia; NEC, necrotizing enterocolitis; Bayley-III, Bayley Scales of Infant and Toddler Development 3rd Edition; NDI, neurodevelopmental impairment

Figure S1. Figure S1. Growth of small and large infants of dichorionic twin (A) and monchorionic twin (B). CA, corrected age.


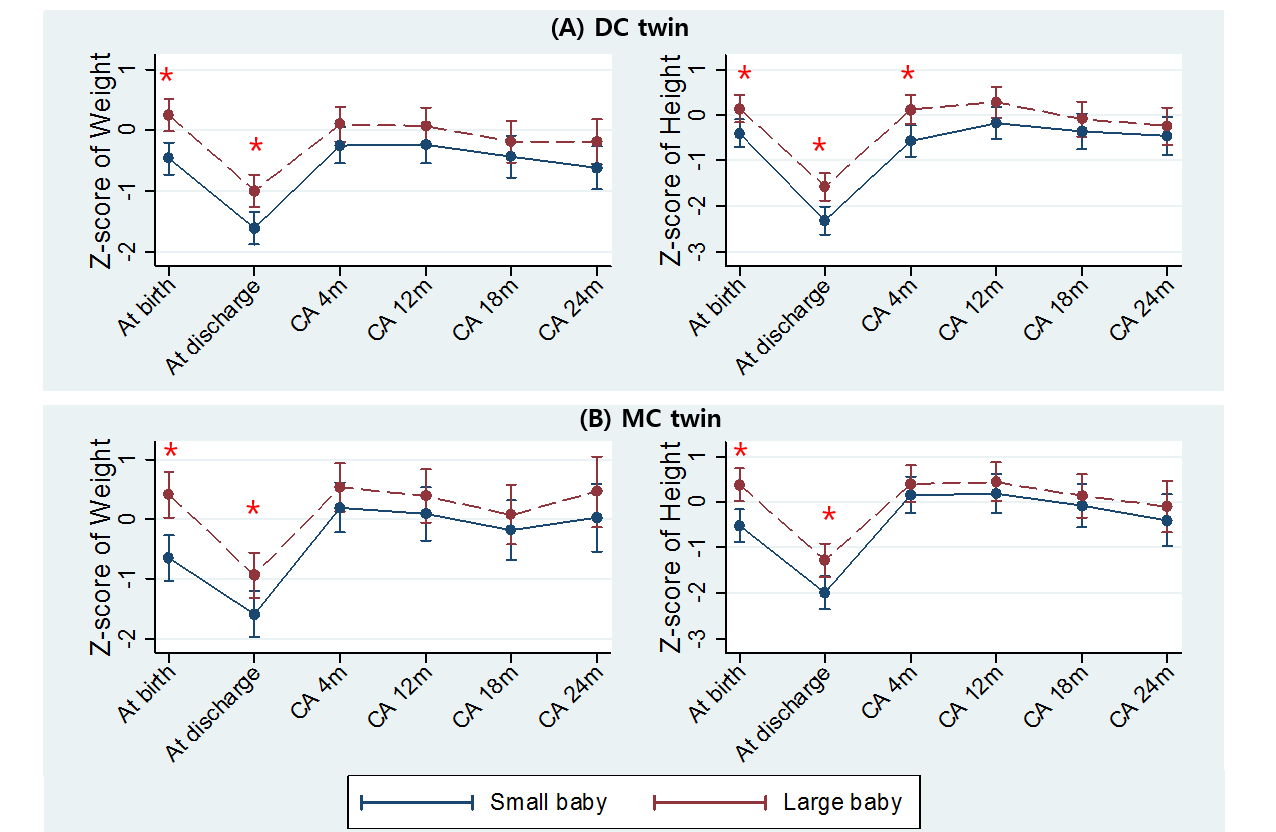

Supplement: Supplementary file 1 — Supplementary Information. [file 41598_2023_33428_MOESM1_ESM.docx]
